# Metabolic Changes in Urine during and after Pregnancy in a Large, Multiethnic Population-Based Cohort Study of Gestational Diabetes

**DOI:** 10.1371/journal.pone.0052399

**Published:** 2012-12-21

**Authors:** Daniel Sachse, Line Sletner, Kjersti Mørkrid, Anne Karen Jenum, Kåre I. Birkeland, Frode Rise, Armin P. Piehler, Jens Petter Berg

**Affiliations:** 1 Department of Medical Biochemistry, University of Oslo, Oslo, Norway; 2 Department of Medical Biochemistry, Oslo University Hospital, Oslo, Norway; 3 Department of Chemistry, University of Oslo, Oslo, Norway; 4 Department of Endocrinology, Morbid Obesity and Preventive Medicine, Oslo University Hospital, Oslo, Norway; 5 Department of Child and Adolescents Medicine, Akershus University Hospital, Lørenskog, Norway; 6 Department of Endocrinology, Morbid Obesity and Preventive Medicine, University of Oslo, Oslo, Norway; 7 Department of General Practice, University of Oslo, Oslo, Norway; 8 Oslo and Akershus University College of Applied Sciences, Oslo, Norway; 9 Fürst Medical Laboratory, Oslo, Norway; The Ohio State University, United States of America

## Abstract

This study aims to identify novel markers for gestational diabetes (GDM) in the biochemical profile of maternal urine using NMR metabolomics. It also catalogs the general effects of pregnancy and delivery on the urine profile. Urine samples were collected at three time points (visit V1: gestational week 8–20; V2: week 28±2; V3∶10–16 weeks post partum) from participants in the STORK Groruddalen program, a prospective, multiethnic cohort study of 823 healthy, pregnant women in Oslo, Norway, and analyzed using ^1^H-NMR spectroscopy. Metabolites were identified and quantified where possible. PCA, PLS-DA and univariate statistics were applied and found substantial differences between the time points, dominated by a steady increase of urinary lactose concentrations, and an increase during pregnancy and subsequent dramatic reduction of several unidentified NMR signals between 0.5 and 1.1 ppm. Multivariate methods could not reliably identify GDM cases based on the WHO or graded criteria based on IADPSG definitions, indicating that the pattern of urinary metabolites above micromolar concentrations is not influenced strongly and consistently enough by the disease. However, univariate analysis suggests elevated mean citrate concentrations with increasing hyperglycemia. Multivariate classification with respect to ethnic background produced weak but statistically significant models. These results suggest that although NMR-based metabolomics can monitor changes in the urinary excretion profile of pregnant women, it may not be a prudent choice for the study of GDM.

## Introduction

Type 2 diabetes (T2DM) is one of the most challenging health problems in this century, and its prevalence is rising – in a worldwide perspective, it is projected that by 2030 more than 500 million people will suffer from diabetes. The escalating costs threaten the health care system of any nation, and complications associated with the disease are a major cause of disability, reduced quality of life, and death. [1], [2] Gestational diabetes mellitus (GDM) shares pathophysiological similarities with T2DM and accordingly, along with the increase of obesity and T2DM in women of reproductive age, an increase of GDM is observed. [3], [4] GDM is defined as any degree of glucose intolerance with onset or first recognition during pregnancy and increases the risk of adverse pregnancy outcomes, and future development of T2DM in both the mothers and their offspring. [4]–[7] Fortunately, short-term pregnancy outcomes can be improved and the risk of later T2DM reduced through lifestyle intervention, turning the prevention of GDM into a crucial opportunity to positively impact the life and health of mother and child. [3], [8], [9].

The STORK Groruddalen research program [10]–[12] aims to improve the identification of pregnancies at high risk for GDM and other complications in order to reduce adverse short and long-term outcomes for mothers and offspring. The name of the program refers to the bird’s symbolic function and the residential area of the study participants, the ethnically highly diverse Groruddalen region of Oslo, Norway. As part of this larger effort, and since GDM is a disorder of the metabolism, NMR metabolomics was undertaken to characterize changes in the urinary profile during and after pregnancy, and to search in these profiles for novel biomarkers for GDM. In the current literature on metabolic adaptations in pregnancy there is a marked focus on glucose and lipid metabolism, and most reports are concerned with measurements on maternal blood. [13]–[15] Much less is published about the urinary excretion profile, but it is known that the glomerular filtration rate increases in pregnancy, and that especially the excretion of amino acids is elevated, particularly when approaching term. Glucose and other sugars may also be excreted at elevated levels, but not only in conjunction with GDM. [16], [17] The current diagnostic protocols for GDM rely on detecting elevated glucose levels in blood, often only late in the second trimester. [18], [19] Therefore, finding substances (or patterns thereof) non-invasively in the urine profiles that could predict the development of GDM before it manifests itself would provide a highly desirable improvement, and the relatively young discipline of metabolomics claims this to be its strength.

Metabolomics, unlike more compound-specific analyses of clinical chemistry, is an approach that tries to model changes in broad profiles of metabolites and relate them to health and disease states. [20] The most commonly used profiling platforms are mass spectrometry (MS) coupled with gas or liquid chromatography and a range of different ionization techniques, or nuclear magnetic resonance spectroscopy (NMR). [21] In the present study, ^1^H-NMR spectroscopy was chosen because it offers the possibility of measuring a large number of small metabolites with a reasonable sensitivity in the micromolar range [21] while conveniently requiring only little sample preparation and acquisition times of typically not more than a few minutes. [22] In studies involving large sets of spectra the analytical variation of results was shown to be as small as 2%, reflecting an impressive degree of reproducibility. [23].

Metabolomics has been successfully applied to the study of a wide range of diseases in humans and animal models, from type 1 and 2 diabetes to autism, asthma and cancer. [24]–[27] In pregnancy research there has been a focus on preeclampsia and other distinct complications [28], and at least one exploratory study also suggested potential biomarkers for GDM. [29].

In this study we have tested whether urine NMR metabolomics can find biomarkers that identify women at risk of developing GDM in a large, multiethnic prospective cohort study. The analysis yielded a comprehensive overview of urinary metabolite concentrations during and after pregnancy which we report as a secondary objective. We have also studied the influence of ethnic background on the metabolite profile.

## Materials and Methods

### Study Population and Sample Collection

The STORK Groruddalen project has been described in detail previously. [10] Briefly, 823 healthy, pregnant women, 59% from ethnic minorities, attending the Child Health Clinics in Groruddalen, Oslo, between 2008 and 2010 were included in the study. Women with diabetes diagnosed before pregnancy were excluded in order to particularly study GDM. The participation rate was 74%, and the participating women were found representative of the main ethnic groups. The participants were on average (± SD) 29.9 (±4.8) years of age, had a prepregnant BMI of 24.6 (±4.8) and were mostly nulli- or uniparous (45.7% and 34.4%, respectively). Previous publications discusses the characteristics and representativeness of the cohort in detail, with a particular focus on ethnic background. [10], [11].

Fasting morning midstream clean-catch urine samples were collected at three visits (V1: gestational week 8–20; V2: week 28±2; V3∶10–16 weeks post partum), and routine tests for nitrite, proteinuria and glucosuria were performed using dipsticks. The remainder was aliquoted and stored at −80°C. Albumin and creatinine concentrations were determined using one of each samples’ aliquots while another was reserved for the NMR analysis described in the present article. At visit V2 a 75 g oral glucose tolerance test (OGTT) was performed, measuring fasting (FPG) and 2-hour venous plasma glucose (2-h PG). The participants who were diagnosed with GDM according to WHO definitions (see below), the current standard in clinical practice in Norway, received lifestyle advice and were remitted to their GP or specialist care for follow-up, where few required insulin. Note that this would only influence a small number of observations at the last visit V3, since V1 and V2 were completed before the diagnosis.

### Definition of Endpoints

GDM was diagnosed independently according to two separate sets of criteria: The first set are the criteria of the World Health Organization (WHO) which define GDM as FPG ≥7.0 or 2-h PG ≥7.8 mmol/L. [19] The second, more finely graded criteria define participants with FPG <5.1 and 2-h PG <8.5 mmol/L as healthy (introducing the abbreviation G0), those above at least one of the limits as having GDM with mild hyperglycemia (G1), and finally those with FPG ≥5.8 mmol/L or 2-h PG ≥11.1 mmol/L as GDM with pronounced hyperglycemia (G2). These latter criteria are based on the recommendations by the International Association of Diabetes and Pregnancy Study Groups (IADPSG). [11], [18], [30].

The WHO criteria identified 13% of the STORK participants as GDM cases. The graded criteria find a GDM prevalence of 32%, further subdivided into 26% with mild (G1) and 6% with more pronounced hyperglycemia (G2).

Ethnic origin was defined by country of birth of the participant or her mother, whichever was more relevant [10], and categorized into Europe (n = 379; including North Americans of European descent), South Asia (n = 200), East Asia (n = 44), Middle East (n = 126; including Central Asia and North Africa), Sub-Saharan Africa (n = 62; mainly Somalia) and South America (n = 12).

### Ethics

The women were given oral and written information, available in eight languages, when attending the Child Health Clinics. Participation was based on written consent. The Regional Ethics committee and The Norwegian Data Inspectorate have approved the study protocol of the STORK Groruddalen research program. The Norwegian Directorate of Health accepted the storage of biological material. [10].

### Acquisition of ^1^H NMR Spectra

Urine samples were thawed, and 900 µl of sample were buffered with 100 µl of a KH_2_PO_4_/KOH solution at pH 7.4 in pure D_2_0, containing NaN_3_ to inhibit bacterial growth and Trimethylsilyl propanoic acid (TSP) as a frequency and concentration reference. The buffered samples were centrifuged at 13,400 *g* and 4°C for 5 minutes, and 600 µl were transferred to 5 mm NMR tubes. Proton NMR spectra were acquired at 300.0 K on a Bruker AV 600 spectrometer equipped with a TCI cryoprobe and an automatic sample changer. Of each sample 32 scans were collected into 64k data points using the Bruker “noesygppr1d” sequence with a spectral width of 20.6 ppm, 2.65 s acquisition time and a 4 s relaxation delay. An exponential line broadening of 0.3 Hz was applied and the Fourier-transformed spectra were referenced to the TSP signal. Additionally, one single-scan, pseudo-2D *J*-resolved spectrum was acquired per sample using the “jresgpprqf” sequence.

Of a selection of representative samples, two-dimensional spectra were acquired in order to facilitate compound identification. [31].

Eventually, after accounting for missing samples and removing a small number of low-quality spectra, a total of 1,911 urine profiles from 790 of the 823 participants (667, 671 and 573 from visits V1, V2 and V3, respectively) were eligible for further analysis. Among these were 572 matched pairs between visits V1 and V2, 509 between V2 and V3, and 494 between V1 and V3. There were 454 complete series with high-quality spectra from all three visits.

### Spectral Processing and Analysis

All spectra were preprocessed with an in-house program written in GNU Octave [32], which first performed a zero-order phase correction on the TSP signal and a first-order correction on the aromatic region of the spectrum, and then subtracted separate linear baselines from the regions up- and downfield of the water artifact. The latter is necessary because the AV 600 instrument lacks an option that produces flat baselines. Finally, the urine spectra were re-referenced to the TSP signal at 0.0 ppm and clipped to the spectral range between -0.5 and 9.0 ppm.

Further processing and analysis was carried out with the statistics environment R. [33] The spectra were first normalized to the area under the TSP signal. An adaptive, nonlinear baseline was then subtracted using the Barkauskas-Xi-Rocke (BXR) algorithm implemented in the R package “FTICRMS”. [34], [35] The water artifact, the TSP signal and the urea region of the spectra were subsequently deleted.

Metabolites were identified by using published literature [36]–[39] and the Human Metabolome Database (HMDB) [40], [41] and were quantified by comparing the area under their respective signals with that of TSP. A number of consistent but unidentified signals were also measured, but their concentrations are consequently relative to an unknown number of contributing protons and therefore reported in arbitrary units. Together, these resulted in a set of ca. 50 concentration variables (or quasi-concentrations, in the case of the unknowns) per sample – a compressed, noise-reduced representation of its spectrum.

Finally, both the spectra and the concentration variables were individually normalized to the absolute creatinine concentration of the respective urine sample. As an internal consistency check, the creatinine concentrations as determined by NMR were compared with measurements performed on a Roche Modular (Roche Diagnostics Ltd., Burgess Hill, UK) at the Central Laboratory, Oslo University Hospital, Aker, and found to be in good agreement (R^2^>95%). Citrate concentrations of a small number of representative samples were also validated against enzymatic measurements [42] at the same laboratory (R^2^>98%). Finally, the distribution of the concentration variables proved to be right-tailed. Therefore, in most analyses the variables were log-transformed, and the results transformed back as necessary.

The urine spectra and the concentration variables were then subjected to principal component analysis (PCA, implemented in the R package “pcaMethods” [43]) in order to survey the most defining variations in the spectra and to search for outliers. Unit-variance scaling was applied in order to amplify the contribution of small signals, i.e. lower-concentration metabolites. The PCA scores plots were color-coded according to the three visits.

To investigate the relations between the spectra and given endpoints, i.e. classifications by visit, diagnosis of GDM, or ethnic background, partial least-squares regression (PLS, using the R package “pls” [44]) was employed in the form of discriminant analysis (PLS-DA). A simple vector of class codes was used when two classes were involved, and a dummy matrix when more than two classes were to be modeled. The analyses were carried out using unit-variance scaling of the variables, and segment-wise cross validation to avoid overfitting. The final models were evaluated by the parameters R^2^, describing the goodness of fit, and more importantly Q^2^, estimating the predictive power after cross validation. Consequently, the ratio Q^2^/R^2^ describes the reliability of a model under cross-validation – whether it performs equally well on new input as on the data it was constructed from. A ratio below 0.5 would raise suspicions of overfitting, i.e. modeling noise in absence of systematic variations, while a model with a Q^2^/R^2^ ratio above 0.8 would be considered consistent and valid. Additionally, the number of misclassifications (NMC) of the PLS-DA models was calculated based on 500 repetitions of the cross validation as an alternative measure of reliability and compared to the distribution of randomly permutated classifications. [45].

Univariate statistical summaries and tests were performed based on the creatinine-normalized, log-transformed concentration variables to support and expand upon any multivariate modeling. In particular, median concentrations and the interquartile range (IQR) of their distributions were calculated for all classes and groups encountered throughout this article, and presented in tables. Two-sample t-tests (or k-sample ANOVAs, as appropriate) were carried out to estimate the significance of group differences. As a special case, when comparing the progression of matched pairs of samples between the three visits, individual fold-change factors could be computed from the log differences, and paired instead of two-sample t-test were appropriate. All p-values are reported without correction for multiple testing.

## Results

### Difference between Visits

**Figure 1:**
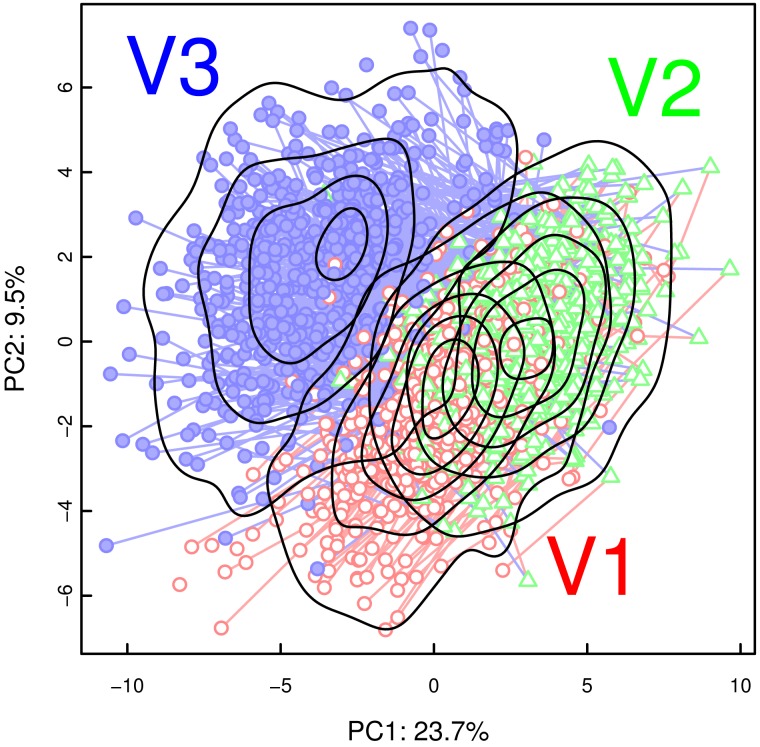
Urine samples distinguish time points during and after pregnancy. PCA scores plot from creatinine-normalized, log-transformed concentration variables, showing the first and second principal component, i.e. the two linear combinations of the original variables that contain the largest and second-largest overall variation (24% and 10%, respectively). Note the clustering of samples from the three visits (V1, gestational week 8–20: red circles; V2, week 26–30: green triangles; and V3, 10–16 weeks post partum: filled blue circles). Red lines connect corresponding samples from visits V1 and V2; blue lines from V2 and V3. Solid black lines represent the density of the scores from the three visits. The overlap between visit V2 and V3 appears to be the smallest.

PCA of the urine spectra produced a weak clustering of samples from the three visits, respectively, but suffered from noise due to e.g. positional variations of compound resonances (data not shown). A PCA of the creatinine-normalized, log-transformed concentration variables, shown in Figure 1, yielded a much clearer picture. There was a certain overlap between the samples from the first and second visit, but in particular the second and third visits were well separated.

The classification potential was demonstrated by PLS-DA on a dummy matrix of all three visits simultaneously: Using 5 components, the model based on the concentration variables correctly classified 86% of the samples compared to 31% in the permutation test. The model based on the spectra, using 7 components, achieved a very similar 85% correct classification.

**Figure 2:**
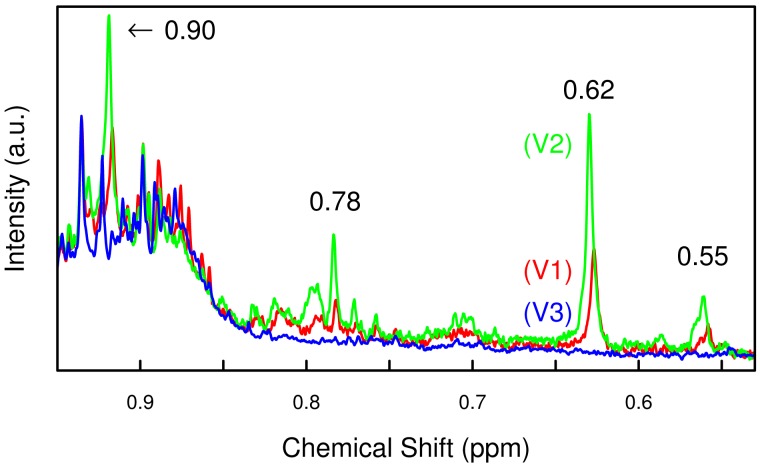
Development of four influential NMR signals over time. Proton NMR spectra of all three urine samples from one healthy participant, showing the region between 0.5 and 0.9 ppm; normalized to the creatinine concentration, BXR baseline correction not yet applied in order to preserve the shape of the broader peaks. The four highlighted signals increase from visit V1 (red line, gest. week 8–20) to V2 (green line, gest. week 26–30) and then disappear at V3 (blue line, 10–16 weeks post partum).

**Figure 3:**
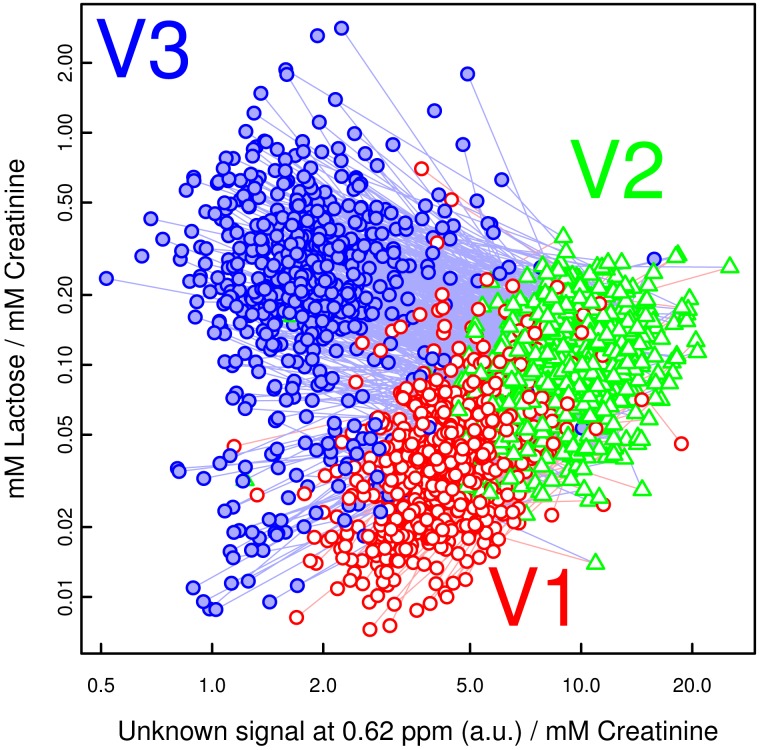
Lactose and an unidentified compound dominate the urinary changes during pregnancy. Scatter plot of concentrations (relative to creatinine concentration; log axes) of lactose and an unidentified substance with an NMR signal at 0.62 ppm. Red circles, green triangles and filled blue circles for visit V1 (gestational week 8–20), V2 (gestational week 26–30) and V3 (10–16 weeks post partum), respectively. Note how these two compounds alone reproduce a clustering similar to that in Fig. 1.

In order to track the specific changes between the visits, pairwise PLS-DA was carried out using both the spectra and the concentration values. The results of the latter are shown in Table 1. As in the PCAs, the most substantial difference happened between visit V2 (late pregnancy) and V3 (post partum), driven primarily by the dramatic reduction of the as yet unidentified compound(s) with NMR resonances at 0.55, 0.62 and 0.78 ppm (Fig. 2), as well as 1.08 and 1.11 ppm. A scatter plot of one of these signals against lactose concentrations (Fig. 3) closely mirrored the clustering found in Fig. 1 using PCA.

**Table 1:** Validation results and most influential compounds of pairwise PLS-DA models from the log-transformed concentration variables with respect to the three visits.

Visit	Q^2^ and R^2^ values of thefirst 3 components^a^	NMC at 5 componentsand permutation test^b^	Involved compounds^c^
V1 → V2	0.526, 0.663, 0.679/0.534, 0.676, 0.700	89.3 (84.0–93.8)/662 (625–695)	Unknown, 0.62 ppm↑; Unknown, 0.78 ppm↑; Alanine↑; Glycine↑; Lactose↑
V2 → V3	0.791, 0.868, 0.883/0.794, 0.873, 0.891	9.0 (8.0–10.0)/607 (581–633)	Unknown, 0.62 ppm↓; Unknown, 0.78 ppm↓; Alanine↓; Glycine↓; Lactose↑
V1 → V3	0.690, 0.782, 0.803/0.695, 0.788, 0.815	35.5 (32.3–39.0)/601 (578–627)	Unknown, 0.62 ppm↓; Unknown, 0.78 ppm↓; Alanine↓; Glycine↓; Lactose↑

a A high ratio between Q^2^ and R^2^ confirms the validity of the models.

b The number of misclassifications (NMC) of the PLS-DA models relative to the random result from permutation testing serves as a performance estimate; 95% CI of the estimates in parentheses.

c Spectral signals that could not be assigned to known metabolites are referred to as “Unknown”, along with the locations of their NMR signals. Arrows denote relative increase or decrease between visits. See also Table 2 and Fig. 2.

Finally, univariate statistics were employed to quantify the changes. Table 2 presents the group-wise median urinary concentrations (with IQR) of selected compounds and unidentified substances relative to the creatinine concentration at the three visits, along with the mean fold change between the visits calculated at the individual level.

**Table 2:** Median concentrations (IQR) of profiled compounds relative to creatinine concentration at the three visits, and patient-wise fold-change between visits.

	Median concentration^a^ (IQR) [mM/mM Creatinine]			Rel. individual FC^a^ (t-test p-value)	
Compound	Visit V1^b^ (<20 wk)	Visit V2^b^ (28 wk)	Visit V3^b^ (3 mo p.p.)	V1 → V2	V2 → V3
Ureac,d	2.5 (1.9–3.3)	2.4 (1.9–3.1)	2.6 (2–3.3)	−1.01 (0.64)	1.07 (0.0025)
Unknown multiplet, 0.55 ppmx,d	2.3 (1.9–2.9)	4.9 (4.1–5.9)	1.7 (1.3–2.2)	2.12 (7e-200)	−2.88 (2.7e-194)
Unknown multiplet, 0.62 ppmx,d	4.2 (3.4–5.3)	9.4 (7.9–11)	1.8 (1.4–2.3)	2.2 (1.4e-219)	−5.14 (2.8e-283)
Unknown multiplet, 0.78 ppmx,e	0.2 (0.091–0.34)	1.2 (0.92–1.6)	0.026 (0–0.084)	6.11 (1.4e-151)	−21.8 (1e-125)
Unknown multiplet, 0.90 ppmx,e	0.28 (0–1.1)	2.6 (1.3–4.4)	0 (0–0.3)	3.42 (1.1e-42)	−4.35 (8e-24)
Unknown doublet, 0.75 ppmx,e	0.047 (0.0085–0.15)	0.04 (0.006–0.13)	0.055 (0.01–0.17)	−1.12 (0.22)	1.45 (0.00027)
Valine	0.0059 (0.004–0.008)	0.006 (0.004–0.008)	0.0041 (0.003–0.006)	−1.02 (0.29)	−1.45 (2.5e-34)
Leucine	0.004 (0.003–0.005)	0.0045 (0.003–0.006)	0.0025 (0.002–0.004)	1.12 (2.1e-11)	−1.83 (8.2e-89)
Unknown doublet, 1.08 ppm^x^	3.5 (2.3–5)	5.4 (3.6–8.1)	2.1 (1.4–3.1)	1.61 (5.9e-68)	−2.63 (1.4e-106)
Unknown doublet, 1.11 ppm^x^	11 (7.5–16)	19 (14–25)	2.9 (1.9–4.4)	1.78 (3.1e-114)	−6.66 (1.2e-231)
3-Aminoisobutyrate^e^	0.0098 (0.004–0.022)	0.011 (0.0041–0.024)	0.008 (0.003–0.019)	1.08 (0.14)	−1.28 (0.00018)
Unknown doublet, 1.24 ppmx,e	2.9 (2.2–4)	4.4 (3–6.2)	3.2 (2.3–4.5)	1.43 (1.6e-38)	−1.33 (2.8e-20)
Unknown doublet, 1.26 ppmx,e	1.3 (0.78–1.9)	1.9 (1.3–2.8)	0.88 (0.41–1.4)	1.55 (1.2e-40)	−2.31 (6.2e-65)
3-Hydroxyisovalerate^e^	0.0092 (0.007–0.012)	0.011 (0.0084–0.014)	0.0054 (0.004–0.007)	1.2 (5.8e-28)	−2.1 (7.9e-145)
2-Hydroxyisobutyrate	0.0071 (0.006–0.008)	0.0076 (0.006–0.009)	0.0054 (0.004–0.006)	1.11 (1.0e-14)	−1.44 (2.5e-87)
comb. Lactate/Threonine^x^	29 (20–43)	56 (39–83)	8.7 (5.8–13)	1.89 (1.6e-89)	−6.3 (2.6e-202)
Alanine	0.04 (0.029–0.054)	0.065 (0.046–0.09)	0.02 (0.015–0.028)	1.62 (1.7e-84)	−3.22 (1.5e-191)
Lysine^e^	0.02 (0.011–0.034)	0.016 (0.009–0.026)	0.0093 (0.005–0.016)	−1.26 (4.7e-08)	−1.64 (6.8e-23)
Acetaminophen metabolitesx,e	9.3 (6.5–14)	10 (6.7–14)	5.8 (3.5–9.4)	1.04 (0.27)	−1.56 (1.1e-14)
Acetone	0.003 (0.002–0.005)	0.004 (0.003–0.006)	0.003 (0.002–0.005)	1.1 (0.38)	−1.09 (0.49)
N-Acetylglutamine	0.055 (0.04–0.08)	0.048 (0.03–0.07)	0.07 (0.045–0.09)	−1.29 (1.5e-13)	1.6 (5.6e-34)
Unknown singlet, 2.35 ppm^x^	17 (9.4–28)	16 (8.1–26)	21 (11–31)	−1.13 (0.00072)	1.3 (3.7e-11)
Citrate	0.24 (0.18–0.3)	0.25 (0.18–0.32)	0.15 (0.1–0.21)	1.03 (0.077)	−1.64 (2.7e-56)
Dimethylamine	0.041 (0.037–0.046)	0.047 (0.043–0.052)	0.042 (0.038–0.048)	1.14 (1.9e-18)	−1.08 (1.8e-06)
Unknown singlet, 2.78 ppm^x^	3 (2.4–3.9)	2.9 (2.4–3.9)	3.6 (2.9–4.8)	−1.01 (0.68)	1.26 (6.8e-17)
Trimethylamine N-oxide	0.038 (0.025–0.064)	0.037 (0.022–0.064)	0.041 (0.027–0.073)	−1.12 (0.019)	1.22 (0.00055)
Glycine	0.24 (0.17–0.36)	0.32 (0.23–0.45)	0.11 (0.056–0.18)	1.32 (3.9e-41)	−3.06 (6.6e-118)
Creatine	0.079 (0.033–0.15)	0.086 (0.042–0.16)	0.05 (0.021–0.13)	1.16 (0.00022)	−1.56 (2.7e-17)
Creatinine^f^ [abs. mM]	10 (7.3–14)	8.5 (6.3–12)	12 (8.8–16)	−1.19 (8.6e-13)	1.33 (1.5e-25)
Trigonelline	0.011 (0.0066–0.018)	0.012 (0.0068–0.023)	0.014 (0.0073–0.027)	1.11 (0.017)	1.14 (0.012)
Lactose	0.04 (0.025–0.06)	0.1 (0.073–0.14)	0.24 (0.14–0.39)	2.47 (6.4e-122)	2.11 (2.8e-51)
1-Methylnicotinamide	0.009 (0.007–0.012)	0.013 (0.0093–0.017)	0.0053 (0.003–0.008)	1.41 (4.3e-42)	−2.58 (5.4e-111)
Unknown singlet, 4.51 ppmx,e	0 (0–0.062)	0 (0–0.25)	0 (0–0.17)	1.1 (0.66)	−1.41 (0.14)
Ascorbate^e^	0.006 (0.004–0.011)	0.01 (0.006–0.016)	0.006 (0.004–0.011)	1.58 (9.6e-14)	−1.47 (6.9e-10)
Glucose	0.1 (0.079–0.13)	0.14 (0.11–0.18)	0.079 (0.064–0.11)	1.39 (6.3e-35)	−1.77 (2.3e-64)
Acetaminophen glucuronide^e^	0.033 (0.023–0.046)	0.039 (0.028–0.053)	0.029 (0.022–0.043)	1.18 (3.9e-08)	−1.18 (1.3e-05)
Unknown multiplet, 5.02 ppmx,e	0.89 (0.43–1.8)	0.93 (0.46–1.8)	1 (0.54–1.9)	1.07 (0.18)	1.12 (0.044)
Unknown doublet, 5.08 ppmx,e	0.089 (0–0.26)	0.084 (0–0.29)	0.12 (0–0.29)	−1.02 (0.8)	1.02 (0.87)
Unknown doublet, 5.20 ppm^x^	1.8 (1.3–2.5)	2.5 (1.9–3.6)	1.2 (0.86–1.7)	1.38 (6.3e-34)	−2.1 (1.2e-75)
Sugar doublets, 5.23 ppm^x^	7.8 (6.1–10)	16 (12–20)	22 (15–33)	1.98 (4.6e-120)	1.27 (3.9e-11)
Unknown doublet, 5.30 ppmx,e	0.53 (0.34–0.81)	1.2 (0.71–1.7)	0.66 (0.47–0.93)	1.99 (7.6e-78)	−1.61 (1.1e-45)
Unknown doublet, 5.42 ppmx,e	0.29 (0.077–0.83)	0.52 (0.18–1.2)	0.42 (0.12–1.1)	1.56 (3.3e-07)	−1.12 (0.22)
1,6-Anhydroglucose^e^	0.003 (0.001–0.006)	0.005 (0.002–0.01)	0.003 (0.002–0.007)	1.64 (1.1e-14)	−1.4 (2.3e-08)
Unknown doublet, 6.58 ppmx,e	0.015 (0–0.17)	0.013 (0–0.18)	0.027 (0–0.22)	1.0 (0.99)	1.08 (0.59)
4-Hydroxyphenylacetate	0.009 (0.007–0.01)	0.01 (0.08–0.014)	0.01 (0.08–0.014)	1.07 (0.028)	−1.01 (0.72)
Tyrosine	0.012 (0.008–0.016)	0.013 (0.0092–0.019)	0.0051 (0.003–0.008)	1.16 (1e-12)	−2.9 (1.2e-91)
N-Phenylacetylglycine^g^	0.074 (0.047–0.11)	0.065 (0.038–0.099)	0.09 (0.054–0.13)	−1.15 (0.00018)	1.37 (3e-14)
Acetaminophen sulfate^e^	0.03 (0.023–0.039)	0.033 (0.025–0.042)	0.03 (0.023–0.04)	1.09 (0.00011)	−1.01 (0.72)
Hippurate	0.21 (0.13–0.32)	0.22 (0.14–0.33)	0.23 (0.14–0.36)	1.02 (0.49)	1.01 (0.68)
Histidine region^g^	0.058 (0.052–0.065)	0.068 (0.061–0.075)	0.053 (0.048–0.06)	1.17 (1.2e-76)	−1.26 (6.3e-101)
Formate	0.035 (0.026–0.052)	0.052 (0.038–0.073)	0.016 (0.011–0.025)	1.51 (1.9e-59)	−3.28 (3.4e-147)

a While the median (IQR) values are reported on their natural scale, all parametric statistical tests were carried out using the log-transformed, normally distributed variables.

b Visit V1: gestational week 8–20; V2: gestational week 26–30; V3∶10–16 weeks post partum.

c Urea is affected by NMR water suppression.

d Broader spectral signal; measured before subtracting BXR baseline.

e Compound very dilute to undetectable in a substantial number of samples; reported concentration may represent noise.

f Concentration of creatinine is reported as absolute mM before normalization.

g No conclusive identification, quantified as stated.

x Concentrations of unidentified signals in arbitrary units, but nonetheless individually normalized to creatinine.

### Gestational Diabetes

The multivariate analysis with respect to GDM yielded few positive results. All Q^2^ were negative for both sets of diagnostic criteria, with the following exceptions: PLS-DA of the concentration variables according to the WHO criteria resulted in barely positive Q^2^ = 2% at visit V1, with the unidentified signal at 1.11 ppm and citrate contributing most to the loading weights. Using a dummy matrix of the graded criteria, PLS-DA yielded Q^2^ = 2% for the pronounced hyperglycemia class G2 at visit V2, involving citrate, glucose and the unidentified signal at 1.08 ppm.

A subsequent univariate analysis supported and quantified these findings. Table 3 shows compounds that were significantly altered at visit V1 and V2, respectively, using t-tests with respect to the two classes of the first, i.e. WHO criteria, along with their median concentrations in said classes. Table 4 shows the same for ANOVA with respect to the three classes of the second, i.e. graded criteria. No significant differences were observed at visit V3 using either diagnostic criteria.

**Table 3:** Selected substances and signals differing between the WHO classes (healthy, diabetes) at visits V1 and V2.

		Median concentration (IQR) [mM/mM Creatinine]		t-test
Visit^a^	Compound	WHO: healthy	WHO: GDM	p-value
V1	Citrate	0.23 (0.18–0.29)	0.28 (0.19–0.34)	2e-4 (↑)
	Unknown doublet, 1.08 ppm^x^	3.4 (2.2–4.8)	4 (2.5–6.4)	0.007 (↑)
	Unknown doublet, 1.11 ppm^x^	11 (7.5–15)	14 (8.9–18)	9e-4 (↑)
V2	Citrate	0.24 (0.17–0.31)	0.3 (0.22–0.4)	7e-5 (↑)
	Unknown doublet, 1.08 ppm^x^	5.4 (3.5–7.9)	5.6 (4.2–10)	0.01 (↑)
	Unknown doublet, 1.11 ppm^x^	19 (14–25)	24 (17–29)	0.001 (↑)

a Visit V1: gestational week 8–20; V2: gestational week 26–30.

x Concentrations of unidentified signals in arbitrary units, but nonetheless normalized to creatinine.

**Table 4:** Selected substances and signals differing between the three graded classes (healthy, GDM with mild hyperglycemia, GDM with pronounced hyperglycemia) at visits V1 and V2.

		Median concentration (IQR) [mM/mM Creatinine]			ANOVA
Visit^a^	Compound	healthy (G0)^b^	GDM, mild (G1)^b^	GDM (G2)^b^	p-value
V1	Glucose	0.1 (0.078–0.13)	0.1 (0.083–0.14)	0.13 (0.096–0.19)	6e-6
	Lysine	0.019 (0.01–0.031)	0.025 (0.012–0.039)	0.026 (0.018–0.044)	4e-4
	Citrate	0.23 (0.18–0.29)	0.24 (0.17–0.32)	0.29 (0.21–0.38)	0.04
	Unknown doublet, 1.08 ppm^x^	3.3 (2.1–4.7)	3.9 (2.5–5.2)	4.4 (2.9–6.8)	9e-4
	Unknown doublet, 1.11 ppm^x^	11 (7.6–15)	12 (8.1–17)	14 (9.1–15)	0.01
V2	Glucose	0.14 (0.11–0.18)	0.14 (0.1–0.19)	0.18 (0.15–0.27)	6e-6
	Citrate	0.24 (0.17–0.31)	0.26 (0.19–0.34)	0.35 (0.29–0.45)	3e-5
	Unknown doublet, 1.08 ppm^x^	5.3 (3.4–7.7)	5.6 (4–8.4)	7.8 (4.5–13)	2e-4
	Unknown doublet, 1.11 ppm^x^	19 (14–25)	19 (15–26)	21 (16–30)	0.03

a Visit V1: gestational week 8–20; V2: gestational week 26–30.

b Classification by graded criteria based on modified IADPSG definitions and the HAPO study: Healthy, normoglycemic (G0); GDM with relatively mild hyperglycemia (G1) or with more pronounced hyperglycemia (G2). Thresholds are defined in the Materials section.

x Concentrations of unidentified signals in arbitrary units, but nonetheless normalized to creatinine.

**Figure 4:**
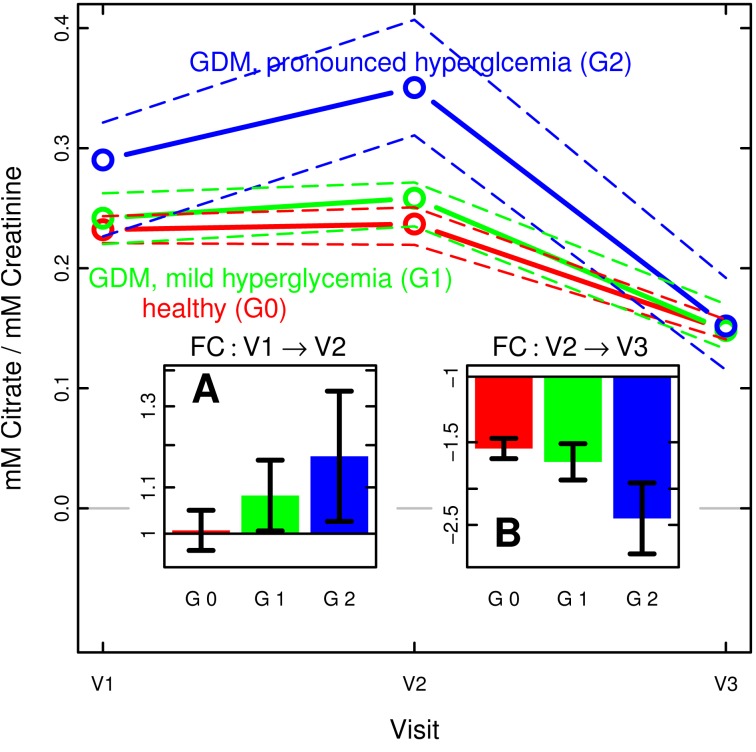
Citrate concentration and relative change during and after pregnancy, by degree of hyperglycemia. Median concentration (±95% CI of the median as dashed lines) of urine citrate concentration relative to creatinine levels at the three visits (V1: gestational week 8–20; V2: gestational week 26–30; V3∶10–16 weeks post partum), shown separately in red, green and blue, respectively, for the three graded classes based on modified IADPSG definitions and the HAPO study (G0: healthy, normoglycemic; G1: GDM with relatively mild hyperglycemia; G2: GDM with more pronounced hyperglycemia). Insets show the mean patient-wise relative fold-change (±95% CI of the mean, based on log values) between visits V1 and V2 (panel A), and V2 and V3 (panel B), respectively. Note the sharper rise and subsequent fall of urinary citrate associated with the severity of GDM.

Expanding on the differences observed for the graded classes, Figure 4 illustrates the development of urine citrate concentration in the three classes during and after pregnancy, presenting the class-wise mean concentration relative to creatinine concentration. The insets show the mean values of the individual fold-changes between the visits of each patient. Clearly, the GDM patients with more pronounced hyperglycemia (G2) exhibited a stronger increase during pregnancy between visit V1 and V2, followed by a steeper decrease after delivery.

### Ethnic Background

PLS-DA using a dummy matrix of ethnic background categories (excluding the very small number of South Americans) was carried out at the three visits and resulted in overall significant models. However, a closer inspection of the cross-validated predictions (see Table 5) revealed that this was mostly driven by the samples from participants with Western background, and potentially South Asians. According to the PLS loading weights, formate, alanine and the combined lactate and threonine variable contributed most to the models, along with 3-hydroxyisovalerate, 1,6-anhydroglucose and an unidentified compound at 0.55 ppm. Note that the latter was also observed above with respect to the progression of pregnancy.

**Table 5:** PLS-DA validation results for dummy matrices of ethnic background at the three visits.

Group^a^	Visit	Q^2^, R^2^ at6 comp.	NMC, permutation test at 6 comp.
West	V1	0.305/0.392	69 (63–74)/161 (150–174)
	V2	0.330/0.410	77 (73–83)/165 (150–179)
	V3	0.317/0.404	77 (72–82)/138 (126–146)
S.Asia^b^	V1	0.108/0.270	109 (105–116)/122 (111–130)
	V2	0.112/0.242	122 (118–126)/121 (114–130)
	V3	0.210/0.315	90 (85–95)/105 (97–113)
E.Asia^c^	V1	0.087/0.149	37/35 (32–37)
	V2	0.087/0.162	31/30 (26–31)
	V3	0.066/0.113	34/32 (29–34)
ME.CA.NA.^d^	V1	0.027/0.100	102/85 (80–91)
	V2	0.066/0.164	99 (97–100)/87 (80–94)
	V3	0.038/0.149	84 (83–85)/73 (67–78)
Sub-Sahara	V1	0.036/0.098	52/48 (45–52)
	V2	0.062/0.112	48/44 (41–47)
	V3	0.045/0.128	37/35 (32–37)

a Excluding South America, n = 12.

b S.Asia: South Asia;

c E.Asia: East Asia;

d ME.CA.NA.: Middle East, Central Asia and North Africa.

Using the categories “Western”, “South Asian” and “Other”, follow-up ANOVAs were performed on all concentration variables at all visits. Significant results are shown in Table 6.

**Table 6:** Selected substances and signals differing between categories of ethnic background at visits V1 through V3.

		Median concentration (IQR) [mM/mM Creatinine]			ANOVA
Visit	Compound	West	South Asia	All Others	p-value
V1	Formate	0.03 (0.024–0.04)	0.04 (0.028–0.059)	0.043 (0.029–0.061)	2e-9
	Alanine	0.034 (0.027–0.047)	0.045 (0.032–0.065)	0.044 (0.031–0.058)	5e-8
	3-Hydroxyisovalerate	0.008 (0.0062–0.011)	0.01 (0.0075–0.013)	0.01 (0.0079–0.013)	2e-8
	1,6-Anhydroglucose	0.0035 (0.002–0.009)	0.003 (0.002–0.006)	0.002 (0.001–0.004)	6e-8
	Lactate/Threonine^x^	25 (17–34)	34 (23–57)	36 (23–55)	4e-12
	Unknown multiplet, 0.55 ppm^x^	2.1 (1.8–2.5)	2.4 (1.9–3.1)	2.5 (2–3.1)	7e-9
V2	Formate	0.043 (0.034–0.058)	0.061 (0.042–0.08)	0.066 (0.046–0.094)	1e-20
	Alanine	0.059 (0.042–0.08)	0.073 (0.051–0.093)	0.069 (0.047–0.1)	1e-5
	3-Hydroxyisovalerate	0.0094 (0.008–0.012)	0.012 (0.01–0.016)	0.012 (0.01–0.015)	3e-12
	1,6-Anhydroglucose	0.006 (0.0032–0.012)	0.004 (0.002–0.007)	0.004 (0.002–0.007)	8e-6
	Lactate/Threonine^x^	49 (36–70)	64 (42–90)	62 (43–93)	3e-6
	Unknown multiplet, 0.55 ppm^x^	4.5 (3.9–5.4)	5.4 (4.3–6.4)	5.3 (4.4–6.6)	5e-10
V3	Formate	0.013 (0.0093–0.019)	0.02 (0.014–0.029)	0.02 (0.013–0.029)	2e-11
	Alanine	0.016 (0.013–0.023)	0.024 (0.019–0.031)	0.022 (0.017–0.031)	8e-14
	3-Hydroxyisovalerate	0.0048 (0.003–0.007)	0.006 (0.004–0.008)	0.006 (0.004–0.008)	3e-6
	1,6-Anhydroglucose	0.0041 (0.0017–0.01)	0.003 (0.001–0.006)	0.003 (0.001–0.005)	2e-6
	Lactate/Threonine^x^	6.9 (4.7–11)	9.8 (7.3–14)	11 (7.1–15)	5e-11
	Unknown multiplet, 0.55 ppm^x^	1.5 (1.2–2)	1.9 (1.4–2.3)	1.8 (1.4–2.3)	5e-4

x Concentrations of the unidentified signal and the combined lactate and threonine variable in arbitrary units, but nonetheless normalized to creatinine.

Finally, the previous ANOVAs with respect to GDM according to the graded classes were repeated separately for the aforementioned three ethnic categories. Compared to Table 4, the results became less reliable: Only in the South Asian category did the increase in glucose (visit V1 and V2) as well as citrate and the unknown signal at 1.08 ppm (both at visit V2) remain significant. None of the variables reached significance in the other two ethnic categories. Nonetheless, the mean citrate concentration exhibited the same association with the graded classes, i.e. the presence and severity of hyperglycemia, and the increase-decrease pattern as described above for the combined data set.

## Discussion

Although its primary aim was the detection of patterns in the urinary metabolome related to GDM, the most immediate finding of the metabolomics efforts that are part of the STORK Groruddalen cohort study is that the changes of the composition of urine during and after pregnancy are substantial enough to clearly differentiate between sampling time points.

The most prominent developments were the steady increase of urinary lactose, and the increase-decrease pattern of a number of NMR signals between 0.55 and 1.10 ppm. Resonances in this region have sometimes been associated with bile acids [46], [47], but our own spike-in experiments (data not shown) failed to confirm this. Spectral simulations using the online tools at nmrdb.org [48], [49] suggest that these signals may belong to pregnanediol and estrogens, or more likely their water-soluble sulfates and glucuronides.

The general increase of lactose is a well-known phenomenon and is linked to lactation and the prolactin levels in blood. Appreciable lactose concentrations in urine are usually first observed at the end of the second trimester, between gestational week 20 and 28, followed by a steady increase over the remainder of the pregnancy and another sharp rise in the days after delivery. [50], [51] However, Fig. 3 clearly shows that a number of participants did in fact have lower urinary lactose concentrations post partum (visit V3) than during pregnancy (visit V1 and particularly visity V2). It remains to be determined at a later stage whether this can be correlated to e.g. breastfeeding habits.

Besides lactose and the presumed hormones, many other concentration variables showed statistically significant developments between the visits. However, since the absolute creatinine concentration, which was used for normalization, varied by 20–30% between visits it is not clear which of the smaller changes are specific to pregnancy and which are due to dilution effects. Nonetheless, it is common clinical practice to relate analyte concentrations in individual urine samples to creatinine, and thus doing so facilitates the comparison of our findings with other reports. [52], [53] In pregnancy research in particular, this issue has been brought up in relation with albumin measurements, where the use of creatinine as a reference was deemed appropriate. [54] It has also been reported that the overall creatinine excretion over a 24-h period does not significantly change in pregnancy, further supporting the case for normalization. [55] Finally, even if one were to dismiss variations in metabolite concentrations below 30%, a number of compounds and signals still rise above this threshold, among them the increase and subsequent decrease of alanine and the combined threonine and lactate signal, and the decrease of glycine, tyrosine and formate after birth. [14], [17].

The metabolomics approach has been previously applied in pregnancy research and has successfully identified biomarker candidates [28], however, many studies used more sensitive mass spectrometry platforms instead of NMR. Examples include an improved prediction of pre-eclampsia [56]–[58] or low birth weight [59]. An exploratory study by Diaz et al. [29] profiled second-trimester maternal urine and plasma from several dozen participants with respect to several endpoints including gestational diabetes. Among others, they observed elevated levels of 3-hydroxyisovalerate and 2-hydroxyisobutyrate and an unassigned doublet signal at 1.10 ppm. Allowing for small shift variations, the latter may coincide with the doublets at 1.08 or 1.11 ppm in our study, whereas the former two only exhibited an insignificant increase in our material. However, in a follow-up analysis using untargeted UPLC-MS the same researchers reported no significant correlations with GDM [60], and neither of the studies found the elevated concentrations of citrate that we discovered.

In a broader perspective, type 1 and 2 diabetes have been studied rather extensively. [61] Profiling urine samples by NMR, Salek et al. studied T2DM in db/db mice, Zucker rats and unmedicated human patients. [24] The mouse and rat samples yielded highly significant multivariate classification models, with disease-correlated increases of urinary citrate, DMA and lactate. The human urine samples had a much larger intra-group variation, but nonetheless a significant increase of citrate, DMA, lactate and several amino acids was observed in the diabetes patients.

Most pertinent to our own work, perhaps, are the studies by Zhao et al. [62] and Zhang et al. [63], which addressed pre-diabetic states and the progression of glucose intolerance, respectively. The former succeeded in identifying patients with impaired glucose tolerance using untargeted UPLC-qTOF mass spectrometry of plasma and urine samples. The urine samples performed worse than plasma, but nonetheless led to a predictive PLS-DA model. Of the compounds involved, only hippurate and phenylacetyl-glutamine were visible in our NMR spectra, and we did not observe a decrease as described by Zhao et al. The Zhang study [63] could differentiate between healthy subjects and overt T2DM cases using NMR spectra of plasma, but could not identify the milder cases with impaired glucose regulation.

It has also been reported previously that ethnic and geographic background can have a large effect on the urinary excretion profile as measured by NMR. [64] As part of the INTERMAP study, a multivariate discriminant analysis could correctly classify over 95% of several hundred urine samples from the United States, Japan and southern China, respectively. Our material, lacking the geographic distribution, showed far less predictive power, indicating that ethnic background had only a comparably subtle impact on the urine profiles. It appears that in terms of “nature vs. nurture”, the urine metabolome seems to be determined more by immediate lifestyle and diet than by genes. Regarding GDM, re-analyzing the concentration variables within the separate ethnic categories reproduced roughly the same mean values as the complete data set, but probably due to the lower number of participants in the respective categories most of these did not reach statistical significance.

NMR-based metabolomics has demonstrated only limited usefulness in the study of a condition with, in the majority of cases, only mild metabolic changes, and several factors aggravate the situation: Even though NMR profiling has a high reproducibility even across laboratories, is non-selective, non-targeted and yet quantitative, its lower sensitivity compared to mass spectrometry means that only a subset of the metabolome can be surveyed. Furthermore, disease-associated concentration changes –or patterns thereof– must be larger than unrelated intra-individual and intra-group variations in order to be recognized by univariate or multivariate statistical methods. The type of study influences the amount of such variations: Case-control or animal studies, for example, typically aim for a high degree of homogeneity which facilitates biomarker detection but may limit their generalizability. Our material from a cohort study, on the other hand, gives a more realistic representation of the population at large but consequently contains more biological variation. The fact that urine also exhibits stronger variations in ionic strength than other matrices, leading to noise in the form of peak shift variations, does not simplify matters. We addressed this complication by primarily working with a matrix of concentrations instead of the raw spectra. Note, however, that all multivariate analyses also were carried out on the spectra. They never outperformed the matrix (data not shown). Apart from analytical difficulties, two opposing effects present challenges to the detection of disease states from urine: While diurnal and dietary variation may increase individual variation that is unrelated to the hypothesis being tested, the body’s homeostasis and renal regulation may mask the impact of the disease on the excretion profile. [65]–[67] It seems to be a common observation that studies analyzing both plasma and urine in parallel, see above, find the former to be correlate better with the disease phenotype. [29], [62].

Note, however, that all these considerations do not categorically invalidate urinary NMR metabolomics. The robustness and ease of use of NMR and in particular the non-invasive nature of urine sampling makes this approach attractive. And while, by its very definition, the search for biomarkers cannot guarantee positive results, the successful classification of the three visits clearly demonstrates that this is possible even in the face of the adverse influences listed above.

### Conclusion

The immediate result of the present study is that urine-based NMR metabolomics can differentiate between time points during and after pregnancy and thus track its development, but that it could not identify reliable biomarkers for gestational diabetes mellitus (GDM) in a large, multiethnic population: The pattern of urinary metabolites, at least above micromolar concentrations, is not influenced strongly and consistently enough by the condition. Nonetheless, an increase of excreted citrate correlated with the severity of GDM was observed, that was consistent with earlier findings.
